# Supplementary material for: Stillbirth in term and late term gestations in Stockholm during a 20-year period, incidence and causes
Source: PLoS One. 2021 May 25;16(5):e0251965. doi: 10.1371/journal.pone.0251965 (PMC8148351; doi:10.1371/journal.pone.0251965)
Supplement: S1 Table — a. Maternal and fetal characteristics comparing term stillbirths at GW 37+0-40+6 with term stillbirths at GW 41+0 and onwards between 1998-2004. b. Maternal and fetal characteristics comparing term stillbirths at GW 37+0-40+6 with term stillbirths at GW 41+0 and onwards between 2005-2013. c. Maternal and fetal characteristics comparing term stillbirths at GW 37+0-40+6 with term stillbirths at GW 41+0 and onwards between 2014-2018. (DOCX) [file pone.0251965.s001.docx]

S1a Table: Maternal and fetal characteristics comparing term stillbirths at GW 37+0-40+6 with term stillbirths at GW 41+0 and onwards between 1998-2004.

| **Maternal and fetal characteristics** | **Term Stillbirth before GW 41+0 n=192** | **Term Stillbirth from GW 41+0 n=69** | **P-value** |
| --- | --- | --- | --- |
| Maternal age, years | 31.1 (SD 5.27) | 32.28 (SD 4.87) | 0.095 |
| Maternal age >35 (n,%) | 47 (24.48%) | 27 (39.13%) | 0.031 |
| BMI, kg/m2 | 24.85 (SD 5.48) | 25.05 (SD 5.69) | 0.815 |
| Nullipara (n,%) | 82 (45.3%) | 32 (49.23%) | 0.689 |
| Born in Sweden (n,%) | 64 (69.57%) | 25 (71.43%) | 1 |
| Born in Africa (n,%) | 4 (4.35%) | 2 (5.71%) | 1 |
| Born in Middel East (n,%) | 14 (15.22%) | 5 (14.29%) | 1 |
| Born in South America (n,%) | 1 (1.09%) | 1 (2.86%) | 1 |
| Born in Asia (n,%) | 3 (3.26%) | 1 (2.86%) | 1 |
| Born in Euroupe/USA/Australia (n,%) | 6 (6.52%) | 1 (2.86%) | 0.709 |
| Smoking (n,%) | 14 (7.45%) | 3 (4.35%) | 0.547 |
| Assisted conseption (n,%) | 7 (3.65%) | 2 (2.9%) | 1 |
| Birthweight ≤10e percentilen (n,%) | 74 (39.36%) | 22 (32.84%) | 0.424 |
| Birthweight <-2 SD (n,%) | 50 (26.6%) | 11 (16.42%) | 0.131 |

S1b Table: Maternal and fetal characteristics comparing term stillbirths at GW 37+0-40+6 with term stillbirths at GW 41+0 and onwards between 2005-2013.

| **Maternal and fetal characteristics** | **Term Stillbirth before GW 41+0 n=265** | **Term Stillbirth from GW 41+0 n=66** | **P-value** |
| --- | --- | --- | --- |
| Maternal age, years | 31.81 (SD 5.18) | 32.24 (SD 5.58) | 0.573 |
| Maternal age >35 (n,%) | 80 (30.19%) | 26 (39.39%) | 0.198 |
| BMI, kg/m2 | 25.36 (SD 4.75) | 26.1 (SD 4.75) | 0.278 |
| Nullipara (n,%) | 84 (39.25%) | 29 (50.88%) | 0.153 |
| Born in Sweden (n,%) | 138 (69.35%) | 32 (64%) | 0.578 |
| Born in Africa (n,%) | 16 (8.04%) | 8 (16%) | 0.151 |
| Born in Middel East (n,%) | 22 (11.06%) | 6 (12%) | 1 |
| Born in South America (n,%) | 1 (0.5%) | 1 (2%) | 0.862 |
| Born in Asia (n,%) | 13 (6.53%) | 2 (4%) | 0.734 |
| Born in Euroupe/USA/Australia (n,%) | 9 (4.52%) | 1 (2%) | 0.682 |
| Smoking (n,%) | 6 (2.33%) | 1 (1.52%) | 1 |
| Assisted conseption (n,%) | 5 (1.89%) | 2 (3.03%) | 0.921 |
| Birthweight ≤10e percentilen (n,%) | 72 (27.17%) | 20 (30.3%) | 0.723 |
| Birthweight <-2 SD (n,%) | 37 (13.96%) | 11 (16.67%) | 0.717 |

S1c Table: Maternal and fetal characteristics comparing term stillbirths at GW 37+0-40+6 with term stillbirths at GW 41+0 and onwards between 2014-2018.

| **Maternal and fetal characteristics** | **Term Stillbirth before GW 41+0 n=144** | **Term Stillbirth from GW 41+0 n=22** | ***P*-value** |
| --- | --- | --- | --- |
| Maternal age, years | 32.75 (SD 5.13) | 31.43 (SD 4.76) | 0.24 |
| Maternal age >35 (n,%) | 54 (37.5%) | 5 (22.73%) | 0.267 |
| BMI, kg/m2 | 25.38 (SD 4.11) | 25.28 (SD 4.39) | 0.92 |
| Nullipara (n,%) | 51 (40.16%) | 12 (63.16%) | 0.101 |
| Born in Sweden (n,%) | 53 (43.44%) | 8 (40%) | 0.964 |
| Born in Africa (n,%) | 7 (5.74%) | 3 (15%) | 0.303 |
| Born in Middel East (n,%) | 29 (23.8%) | 2 (10%) | 0.276 |
| Born in South America (n,%) | 2 (1.64%) | 1 (5%) | 0.897 |
| Born in Asia (n,%) | 7 (5.74%) | 1 (5%) | 1 |
| Born in Euroupe/USA/Australia (n,%) | 9 (7.38%) | 3 (15%) | 0.482 |
| Smoking (n,%) | 3 (5.17%) | 0 (0%) | 1 |
| Assisted conseption (n,%) | 9 (6.34%) | 1 (4.55%) | 1 |
| Birthweight ≤10e percentilen (n,%) | 34 (24.29%) | 9 (40.91%) | 0.167 |
| Birthweight <-2 SD (n,%) | 13 (9.29%) | 4 (18.18%) | 0.373 |
