# Supplementary material for: Stillbirth in term and late term gestations in Stockholm during a 20-year period, incidence and causes
Source: PLoS One. 2021 May 25;16(5):e0251965. doi: 10.1371/journal.pone.0251965 (PMC8148351; doi:10.1371/journal.pone.0251965)
Supplement: S2 Table — a. Main cause of stillbirth according to the Stockholm Stillbirth Classification comparing term stillbirths at GW 37+0 -40+6 with term stillbirths at GW 41+0 and onwards between 1998-2004. b. Main cause of stillbirth according to the Stockholm Stillbirth Classification comparing term stillbirths at GW 37+0 -40+6 with term stillbirths at GW 41+0 and onwards between 2005-2013. c. Main cause of stillbirth according to the Stockholm Stillbirth Classification comparing term stillbirths at GW 37+0 -40+6 with term stillbirths at GW 41+0 and onwards between 2014-2018. (DOCX) [file pone.0251965.s002.docx]

S2a Table: Main cause of stillbirth according to the Stockholm Stillbirth Classification comparing term stillbirths at GW 37+0 -40+6 with term stillbirths at GW 41+0 and onwards between 1998-2004.

| **Main cause of stillbirth** | **Term Stillbirth before GW 41+0 n=192** | **Term Stillbirth from GW 41+0 n=69** | **p-value** |
| --- | --- | --- | --- |
| Malformation/chromosomal abnormalities (%) | 18 (9.38%) | 4 (5.8%) | 0.506 |
| Infection | 41 (21.35%) | 29 (42.03%) | 0.002 |
| Feto-maternal transfusion | 7 (3.65%) | 0 (0%) | 0.241 |
| Placental insufficiency/IUGR | 34 (17.71%) | 9 (13.04%) | 0.48 |
| Umbilical cord complications | 21 (10.94%) | 3 (4.35%) | 0.167 |
| Placental abruptio | 21 (10.94%) | 3 (4.35%) | 0.167 |
| Preeclampsia | 5 (2.6%) | 2 (2.9%) | 1 |
| Diabetes mellitus | 3 (1.56%) | 0 (0%) | 0.7 |
| Intrahepatic cholestasis of pregnancy | 1 (0.52%) | 0 (0%) | 1 |
| Coagulation disorder | 0 (0%) | 2 (2.9%) | 0.118 |
| Other causes related to stillbirth | 8 (4.17%) | 3 (4.35%) | 1 |
| Cause of stillbirth un-known | 30 (15.62%) | 12 (17.39%) | 0.88 |

S2b Table: Main cause of stillbirth according to the Stockholm Stillbirth Classification comparing term stillbirths at GW 37+0 -40+6 with term stillbirths at GW 41+0 and onwards between 2005-2013.

| **Main causes of stillbirth** | **Term Stillbirth before GW 41+0 n=265** | **Term Stillbirth from GW 41+0 n=66** | **P-value** |
| --- | --- | --- | --- |
| Malformation/chromosomal abnormalities (%) | 22 (8.3%) | 2 (3.03%) | 0.225 |
| Infection | 48 (18.11%) | 30 (45.45%) | <0.001 |
| Feto-maternal transfusion | 8 (3.02%) | 0 (0%) | 0.327 |
| Placental insufficiency/IUGR | 86 (32.45%) | 20 (30.3%) | 0.851 |
| Umbilical cord complications | 29 (10.94%) | 4 (6.06%) | 0.34 |
| Placental abruptio | 18 (6.79%) | 4 (6.06%) | 1 |
| Preeclampsia | 3 (1.13%) | 0 (0%) | 0.887 |
| Diabetes mellitus | 7 (2.64%) | 1 (1.52%) | 0.932 |
| Intrahepatic cholestasis of pregnancy | 1 (0.38%) | 0 (0%) | 1 |
| Coagulation disorder | 0 (0%) | 2 (2.9%) | 0.118 |
| Other causes related to stillbirth | 4 (1.51%) | 0 (0%) | 0.708 |
| Cause of stillbirth un-known | 33 (12.45%) | 5 (7.58%) | 0.37 |

S2c Table: Main cause of stillbirth according to the Stockholm Stillbirth Classification comparing term stillbirths at GW 37+0 -40+6 with term stillbirths at GW 41+0 and onwards between 2014-2018.

| **Main cause of stillbirth** | **Term Stillbirth before GW 41+0 n=144** | **Term Stillbirth from GW 41+0 n=22** | **P-value** |
| --- | --- | --- | --- |
| Malformation/chromosomal abnormalities (%) | 5 (4.1%) | 1 (5.26%) | 1 |
| Infection | 33 (27.05%) | 6 (31.58%) | 0.893 |
| Feto-maternal transfusion | 5 (4.1%) | 1 (5.26%) | 1 |
| Placental insufficiency/IUGR | 49 (40.16%) | 10 (52.63%) | 0.438 |
| Umbilical cord complications | 5 (4.1%) | 0 (0%) | 0.817 |
| Placental abruptio | 4 (3.28%) | 0 (0%) | 0.954 |
| Diabetes mellitus | 1 (0.82%) | 0 (0%) | 1 |
| Intrahepatic cholestasis of pregnancy | 2 (1.64%) | 0 (0%) | 1 |
| Coagulation disorder | 1 (0.82%) | 0 (0%) | 1 |
| Cause of stillbirth un-known | 17 (13.93%) | 1 (5.26%) | 0.494 |
